# Supplementary material for: Serum Cartilage Oligomeric Matrix Protein and Golgi Protein-73: New Diagnostic and Predictive Tools for Liver Fibrosis and Hepatocellular Cancer?
Source: Cancers (Basel). 2021 Jul 13;13(14):3510. doi: 10.3390/cancers13143510 (PMC8304371; doi:10.3390/cancers13143510)
Supplement: Supplementary file 1 [file cancers-13-03510-s001.zip › cancers-1248014-supplementary.pdf]

Supplementary Material: Serum Cartilage Oligomeric Matrix Protein and Golgi Protein-73: New Diagnostic and Predictive Tools for Liver Fibrosis and Hepatocellular Cancer?

Nikolaos K. Gatselis, Kalliopi Zachou, George Giannoulis, Stella Gabeta, Gary L. Norman and George N. Dalekos

Table S1. Diagnostic performance of GP73, COMP, APRI and FIB-4 and their combinations for detection of cirrhosis or significant fibrosis.

Combinations	AUC (95%CI) for cirrhosis (*n* = 288)	*P*-value	AUC (95%CI) for significant (≥F2) fibrosis based on liver biopsy (*n* = 177)	*P*-value	AUC (95%CI) for significant (≥F2) fibrosis based on TE (*n* = 127)	*P*-value	AUC (95%CI) for significant (≥F3) fibrosis based on liver biopsy (*n* = 177)	*P*-value	AUC (95%CI) for significant (≥F3) fibrosis based on TE (*n* = 127)	*P*-value
GP73 + COMP + APRI + FIB-4	0.916 (0.878–0.946)	ref	0.832 (0.768–0.883)	ref	0.822 (0.744–0.884)	0.239	0.844 (0.783–0.894)	ref	0.806 (0.726–0.871)	0.414
GP73 + COMP + APRI	0.889 (0.846–0.923)	0.01	0.802 (0.736–0.858)	0.129	0.800 (0.720–0.866)	0.078	0.809 (0.743–0.864)	0.049	0.792 (0.710–0.859)	0.270
GP73 + COMP + FIB-4	0.913 (0.874–0.943)	0.578	0.829 (0.765–0.881)	0.775	0.841 (0.766–0.900)	ref	0.841 (0.779–0.892)	0.680	0.817 (0.738–0.880)	ref
GP73 + COMP	0.882 (0.839–0.917)	0.004	0.789 (0.722–0.847)	0.04	0.791 (0.710–0.858)	0.078	0.800 (0.733–0.856)	0.026	0.804 (0.724–0.869)	0.662
GP73 + APRI	0.862 (0.817–0.900)	0.004	0.773 (0.704–0.833)	0.077	0.706 (0.619–0.784)	<0.001	0.782 (0.714–0.840)	0.034	0.726 (0.640–0.801)	0.007
COMP + APRI	0.829 (0.781–0.871)	<0.001	0.755 (0.684–0.816)	0.007	0.809 (0.729–0.873)	0.361	0.780 (0.712–0.839)	0.011	0.771 (0.688–0.841)	0.169
GP73 + FIB-4	0.901 (0.861–0.933)	0.145	0.826 (0.762–0.879)	0.800	0.788 (0.707–0.856)	0.012	0.845 (0.783–0.895)	0.963	0.764 (0.680–0.834)	0.012
COMP + FIB-4	0.895 (0.853–0.928)	0.03	0.801 (0.735–0.857)	0.104	0.840 (0.773–0.905)	0.795	0.834 (0.771–0.886)	0.515	0.805 (0.725–0.870)	0.588
APRI + FIB-4	0.882 (0.839–0.917)	0.005	0.780 (0.711–0.838)	0.027	0.755 (0.671–0.827)	0.021	0.825 (0.761–0.878)	0.386	0.725 (0.639–0.801)	0.014
GP73	0.843 (0.795–0.883)	<0.001	0.742 (0.671–0.804)	0.01	0.676 (0.587–0.756)	<0.001	0.759 (0.689–0.820)	0.009	0.725 (0.638–0.800)	0.033
COMP	0.770 (0.716–0.817)	<0.001	0.708 (0.635–0.774)	<0.001	0.772 (0.690–0.842)	0.100	0.724 (0.652–0.788)	<0.001	0.766 (0.683–0.836)	0.227
APRI	0.833 (0.784–0.874)	<0.001	0.762 (0.692–0.822)	0.068	0.759 (0.675–0.830)	0.052	0.810 (0.745–0.865)	0.288	0.738 (0.653–0.812)	0.043
FIB-4	0.885 (0.846–0.920)	0.02	0.799 (0.732–0.865)	0.171	0.802 (0.722–0.868)	0.210	0.841 (0.778–0.891)	0.865	0.756 (0.672–0.828)	0.038

P-values were calculated by DeLong test, using the combination with the higher AUC as reference. AUC: Area under the curve. GP73: Golgi protein 73. COMP: Cartilage oligomeric matrix protein. APRI: Aspartate aminotransferase to platelets index. FIB-4: Fibrosis-4. REF: reference. Probability p(GP73 + COMP + APRI + FIB-4) = $1/[1 + e^{-(0.116 \times GP73 + 0.128 \times COMP - 0.894 \times APRI + 1.029 \times FIB\text{-}4 - 4.883)}]$. Probability p(GP73 + COMP + APRI) = $1/[1 + e^{-(0.158 \times GP73 + 0.175 \times COMP + 0.447 \times APRI - 4.821}]$. Probability p(GP73 + COMP+FIB-4) = $1/[1 + e^{-(0.117 \times GP73 + 0.137 \times COMP + 0.628 \times FIB\text{-}4 - 4.856}]$. Probability p(GP73 + COMP) = $1/[1 + e^{-(0.177 \times GP73 + 0.180 \times COMP - 4.719)}]$. Probability p(GP73 + APRI) = $1/[1 + e^{-(0.168 \times GP73 + 0.570 \times APRI - 3.096)}]$. Probability p(COMP + APRI) = $1/[1 + e^{-(0.193 \times COMP + 0.766 \times APRI - 3.298)}]$. Probability p(GP73 + FIB-4) = $1/[1 + e^{-(0.121 \times GP73 + 0.753 \times FIB\text{-}4 - 3.633)}]$. Probability p(COMP + FIB-4) = $1/[1 + e^{-(0.150 \times COMP + 0.860 \times FIB\text{-}4 - 4.024)}]$. Probability p(APRI + FIB-4) = $1/[1 + e^{-(-1.070 \times APRI + 1.441 \times FIB\text{-}4 - 2.703)}]$.
